# Developing a prognosis and chemotherapy evaluating model for colon adenocarcinoma based on mitotic catastrophe-related genes

**DOI:** 10.1038/s41598-024-51918-7

**Published:** 2024-01-18

**Authors:** Yinglei Liu, Yamin Zhao, Siming Zhang, Shen Rong, Songnian He, Liqi Hua, Xingdan Wang, Hongjian Chen

**Affiliations:** 1 Nantong Tumor Hospital and Affiliated Tumor Hospital of Nantong University, Nantong, China; 2 Affiliated Hospital 2 of Nantong University, Nantong First People’s Hospital, Nantong, China; 3 The Second People’s Hospital of Nantong, Nantong, China

**Keywords:** Bioinformatics, Cancer genomics, Gastrointestinal cancer, Biomarkers, Risk factors

## Abstract

Mitotic catastrophe (MC) is a novel form of cell death that plays an important role in the treatment and drug resistance of colon adenocarcinoma (COAD). However, MC related genes in COAD treatment and prognosis evaluation are rarely studied. In this study, the transcriptome data, somatic mutation and copy number variation data were obtained from The Cancer Genome Atlas (TCGA) database. The mitotic catastrophe related genes (MCRGs) were obtained from GENCARDS website. Differential gene analysis was conducted with LIMMA package. Univariate Cox regression analysis was used to identify prognostic related genes. Mutation analysis was performed and displayed by maftools package. RCircos package was used for localizing the position of genes on chromosomes. “Glmnet” R package was applied for constructing a risk model via the LASSO regression method. Consensus clustering analyses was implemented for clustering different subtypes. Functional enrichment analysis through Gene Ontology (GO) and Kyoto Encyclopedia of Genes and Genomes (KEGG) methods, immune infiltration analysis via single sample gene set enrichment analysis (ssGSEA), tumor mutation burden and drug sensitivity analysis by pRRophetic R package were also carried out for risk model or molecular subtype’s assessment. Additionally, the connections between the expression of hub genes and overall survival (OS) were obtained from online Human Protein Atlas (HPA) website. Real-Time Quantitative Polymerase Chain Reaction (RT‑qPCR) further validated the expression of hub genes. A total of 207 differentially expressed MCRGs were selected in the TCGA cohort, 23 of which were significantly associated with OS in COAD patients. Subsequently, we constructed risk score prognostic models with 5 hub MCRGs, including SYCE2, SERPINE1, TRIP6, LIMK1, and EEPD1. The high-risk patients suffered from poorer prognosis. Furthermore, we developed a nomogram that gathered age, sex, staging, and risk score to accurately forecast the clinical survival outcomes in 1, 3, and 5 years. The results of functional enrichment suggested a significant correlation between MCRGs characteristics and cancer progression, with important implications for the immune microenvironment. Moreover, patients who displayed high TMB and high risk score showed worse prognosis, and risk characteristics were associated with different chemotherapeutic agents. Finally, RT‑qPCR verified the increased expression of the five MCRGs in clinical samples. The five MCRGs in the prognostic signature were associated with prognosis, and could be treated as reliable prognostic biomarkers and therapeutic targets for COAD patients with distinct clinicopathological characteristics, thereby providing a foundation for the precise application of pertinent drugs in COAD patients.

## Introduction

Colon Adenocarcinoma (COAD), the most common histological subtype of Colorectal cancer (CRC), has one of the highest incidences and mortalities among malignant tumors^1,2^. Surgical resection and chemotherapy are the primary treatments^3^, but the adverse effects of chemotherapy can reduce patients’ quality of life and worsen their prognosis^4^. The occurrence and development of COAD is a complex, heterogeneous process that is influenced by genetic variation, cellular and external environmental factors, and no specific carcinogenic mechanism has been identified^4^. Worse still, the majority of COAD patients are metastatic when diagnosed, which results in about 14% possibility on 5-year survival rate although under systemic treatment^5^. At present, the pathogenesis of COAD has not been fully elucidated, so it is very important to find valuable molecular targets^6–8^, which can provide new treatments for patients with COAD.

Mitotic catastrophe (MC) is a newly recognized form of cell death that arises from dysregulation of the mitotic process and functions as an endogenous tumor suppressor mechanism^9^. MC is a complex signal cascade that drives cells to undergo mitosis and may serve as a new target for cancer therapy^10^. In recent years, the mechanism of MC occurrence and development has garnered much attention, and inducing MC in tumor cells has been used to optimize clinical treatment of tumors and reverse multidrug resistance in some tumors^11,12^. For instance, Jung et al.^13^ observed that induction of MC in oral cancer cells, could be a promising treating approach. Other studies also have shown associations between MC and favorable prognosis in colon cancer^9^, breast cancer^14^, and prostate cancer^15^. Despite these findings, the functions of MC-related genes (MCRGs) in the prognosis of COAD patients have not been reported.

In this study, we explored the expression of MCRGs and their prognostic value in COAD patients. The risk model based on selected MCRGs for predicting the prognosis of COAD patients showed better results and provided potential biomarkers for the treatment of COAD patients.

## Methods

### Data acquisition and processing

The transcriptome data of 447 patients with COAD and 41 normal samples and clinical information were downloaded from The Cancer Genome Atlas (TCGA) database (https://portal.gdc.cancer.gov/repository) accompanied with relevant somatic mutation and copy number variation (CNV) data. The MCRGs were obtained from GENCARDS (https://www.genecards.org/) (Table S1) and identified differentially expressed MCRGs between tumor samples and normal samples (Table S2) by the the Bioconductor Linear Model for Microarray Analysis (LIMMA) package (FDR < 0.05, |log2 FC|≥ 2)^16^. Then, univariate Cox regression analysis was used applying coxph function in survival package^17^ to identify differentially expressed MCRGs that were significantly associated with overall survival (OS) (Table S3, *P* < 0.05).

### Mutation analysis of MCRGs

The mutation frequency and oncoplot waterfall plot of 23 prognosis-related MCRGs in COAD patients were generated by the “maftools” package. The R package "ggplot2" was also used to show the frequency of gene gain and loss. The location of CNV alteration of 23 MCRGs onchromosomes was drawn using the “RCircos” package in R.

### Prognostic risk model construction for MCRGs

The five genes with the best prognosis were screened in the TCGA dataset using the LASSO regression in the “glmnet” R package. The risk score model was constructed by linear fitting, where the weight of each gene was determined by the regression coefficient obtained from the LASSO regression analysis.

The TCGA data set was divided into a 50% training set and a 50% testing set applying the function createDataPartition () in the caret R package, and all patients were divided into a high-risk group and a low-risk group according to the median risk score. The predictive accuracy of the risk score model was assessed by using the “survROC” R package for the receiver operating characteristic (ROC) curve, and the area under the curve^18^ was calculated at different time points (year 1, year 2 and year 3). Heat maps for both groups were created using the “pheatmap” R package.

Based on independent clinical characteristics (age, sex, TNM staging and risk signature) explored by univariate and multivariate Cox regression analyses, we constructed a multifactorial prognostic nomogram using the “rms” and “survival” R packages. Verification of the accuracy of the nomogram based on time-dependent ROC curves for 1, 3 and 5 years, predicted calibration curves, and the concordance index (c-index).

### Consensus clustering analyses

Consensus clustering analyses were performed using the k-means algorithm in the “Consensus Cluster Plus” R package. The optimal number of subtypes was determined by assessing the consistent matrix and cumulative distribution function (CDF). Based on the transcription matrix of the five genes in the signature, two robust subtypes were identified. We used Kaplan–Meier analysis in the R “survminer” package to determine whether COAD subtypes exhibited significant survival differences.

### Functional enrichment analysis and immune function status analysis

We used single sample gene set enrichment analysis (ssGSEA) in the Gene Set Variation Analysis (GSVA) package^19^ to calculate 23 types of immune cells infiltration, and detected differences in immune cell infiltrates between different subgroups using the Wilcoxon rank sum test. In addition, the two subtypes were analyzed by Gene Ontology (GO, http://www.geneontology.org/)^20^ and Kyoto Encyclopedia of Genes and Genomes (KEGG, https://www.kegg.jp/kegg/kegg1.html)^21,22^ using the R “clusterprofiler” package^23^.

### Tumor mutation burden correlation

We evaluated the Tumor Mutation Burden (TMB) score of each COAD patient using somatic mutation analysis, and divided patients into low TMB and high TMB groups^24^. The overall survival^25^ of both groups was compared using Kaplan–Meier analysis. Similarly, we divided COAD patients into four groups according to TMB and risk score, and compared the OS of these four groups^24^.

### The connections between MCRGs expression and OS in human protein atlas

To verify the expression and prognostic significance of MCRGs, we searched for hub genes on the Human Protein Atlas (HPA) website (https://www.proteinatlas.org/), and obtained immunohistochemical (IHC) staining images^26^. Then, Kaplan–Meier Plotter website (http://kmplot.com/) was performed to verify the prognosis of hub genes.

### Drug sensitivity analysis

We used the “pRRophetic” R software package to calculate the half inhibitory concentration (IC50) values of tumor therapeutic drugs and evaluate the difference in efficacy between high and low risk groups. We also analyzed the correlation between IC50 and risk score using Spearman's correlation test. The lower IC50 value, the higher treatment sensitivity.

### Collection of clinical specimens

Here, eight pairs of surgically excised colorectal cancer (CRC) and matched normal tissues were procured, immediately cryopreserved in liquid nitrogen, and stored at − 80 °C. None of the colorectal cancer patients had undergone any preoperative anti-tumor therapies. All patients provided informed consent, and the study was approved by the the Ethics Committee of Nantong Tumor Hospital (no. 2022-061). All methods were performed in accordance with the relevant guidelines and regulations.

### Real-time quantitative polymerase chain reaction (RT‑qPCR)

Total RNA was extracted using TRIzol reagent (Invitrogen, Thermo Scientific, Shanghai, China) following the manufacturer's instructions. cDNA was synthesized using the QuantiTect Reverse Transcription Kit (QIAGEN, Valencia, CA, USA), and real-time PCR was performed using SYBR-Green (Takara, Otsu, Shiga, Japan). Relative gene expression levels were analyzed using the 2-ΔΔCt method and normalized to GAPDH. The primer sequences used for RT-qPCR were shown in Table 1. All samples were tested in triplicate.

**Table 1:** The primer sequences in this study.

GAPDH-F	TGACATCAAGAAGGTGGTGAAGCAG
GAPDH-R	GTGTCGCTGTTGAAGTCAGAGGAG
EEPD1-F	GTATGCAGGATTCCTATGGGAC
EEPD1-R	GAAGGTTAACAAGGGTCAGGTC
LIMK1-F	GATGTGAAGAATTCCATCCACG
LIMK1-R	GAATCAGCAGGTCAATCTCGTC
TRIP6-F	CTATAGGAGCCAGAGAGAGCC
TRIP6-R	CTTCTTCGTCAGCCTATCCAG
SERPINE1-F	AACGTGGTTTTCTCACCCTAT
SERPINE1-R	CAATCTTGAATCCCATAGCTGC
SYCE2-F	TCGGGAGGGATAGGAGGGACAG
SYCE2-R	TGGGAGAGGCGGCTTCAGATG

### Statistical analysis

All statistical analyses were performed using R package (version 4.0.4)^23^. The Wilcoxon test was used to compare statistical differences between the two groups. The Spearman test was used to calculate correlation coefficients. The log-rank test was used to determine the significance of Kaplan–Meier analysis of survival differences. *P* values < 0.05 were considered statistically significant.

### Ethics statement

The studies involving human participants were reviewed and approved by the Ethics Committee of Nantong Tumor Hospital (no. 2022-061). The patients provided their written informed consent to participate in this study, complying with the current laws in China.

## Results

### The genetic and expression landscape of prognostic MCRGs in COAD samples

**Figure 1:**
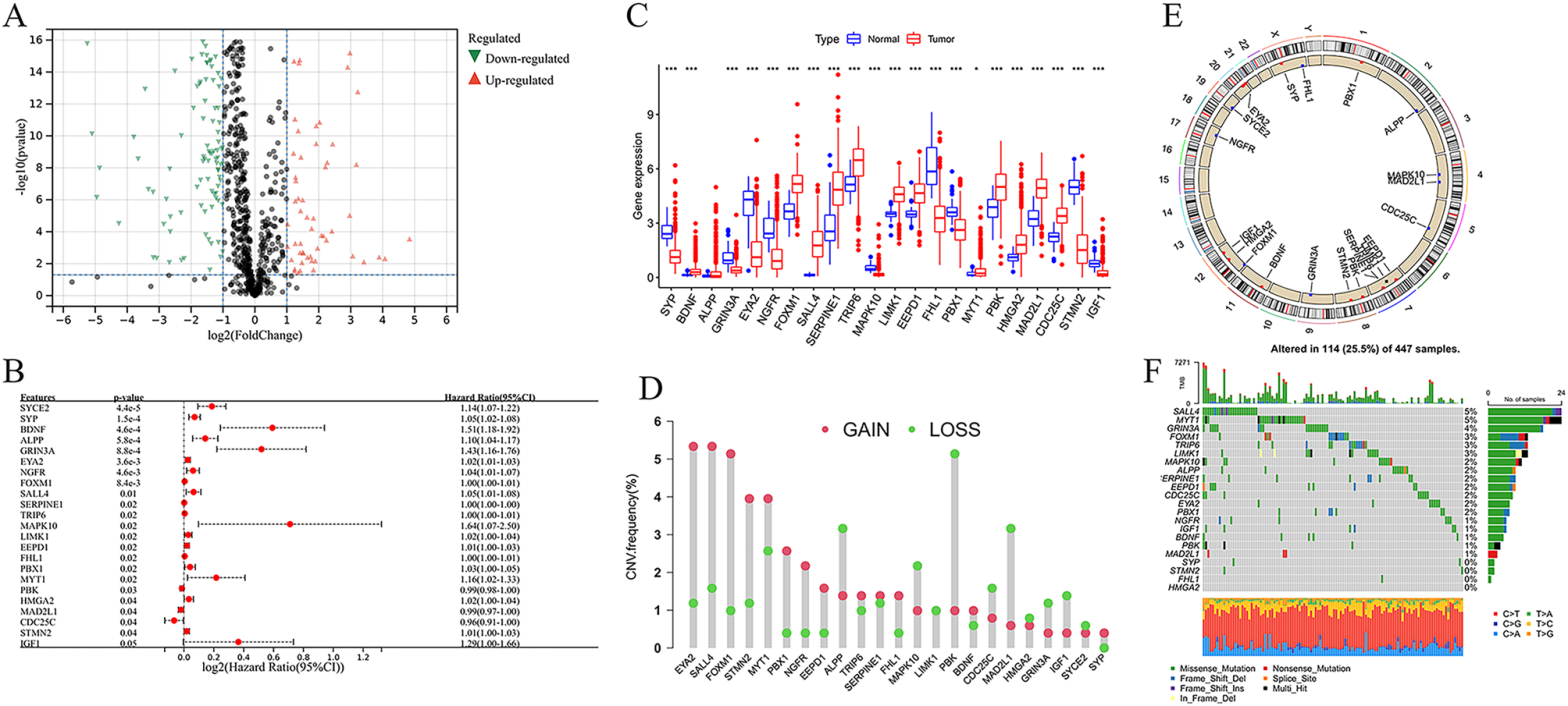
Genetic and expression variation of mitotic catastrophe-related genes (MCRGs) in colon adenocarcinoma (COAD). (**A**) Volcano plot of differentially expressed MCRGs. (**B**) Forest plot of univariate Cox analysis showing the 23 MCRGs significantly associated with OS in colon cancer patients. (**C**) Expression of 23 MCRGs in COAD and normal colon tissues. The upper and lower ends of the boxes indicate the interquartile range of values. Lines in the boxes represent medians. *P < 0.05, ***P < 0.001. (**D**) Copy number variation (CNV) frequencies of the 23 MCRGs in the COAD cohort. (**E**) Location of CNV alterations in 23 MCRGs on 23 chromosomes in the cohort. (**F**) Mutation frequencies and classification of 23 MCRGs in COAD.

To identify prognostic MCRGs in COAD, 900 MCRGs were obtained from GENECARDS (Table S1). Combined with TCGA COAD databases, a total of 207/894 differentially expressed genes were selected in the Fig. 1A (Tables S2 and S3). Among them, SYCE2, SYP, BDNF, ALPP, GRIN3A, EYA2, NGFR, FOXM1, SALL4, SERPINE1, TRIP6, MAPK10, LIMK1, EEPD1, FHL1, PBX1, MYT1, PBK, HMGA2, MAD2L1, CDC25C, STMN2, and IGF1 were identified as candidate genes significantly associated with the OS of COAD patients (Fig. 1B). We examined the expression of these 23 prognostic MCRGs in COAD and normal tissues. Compared with normal tissues, the expression of BDNF, FOXM1, SALL4, SERPINE1, TRIP6, LIMK1, EEPD1, MYT1, PBK, HMGA2, MAD2L1, and CDC25C was increased, while SYP, GRIN3A, EYA2, NGFR, MAPK10, FHL1, PBX1, STMN2, and IGF1 expression was decreased in COAD (Fig. 1C).

We also investigated the frequency of CNV alterations in 23 prognostic MCRGs. The result showed that EYA2, SALL4, FOXM1, STMN2, MYT1, PBX1, NGFR, EEPD1, TRIP6, SERPINE1, FHL1, BDNF, and SYP had copy number amplification. While ALPP, MAPK10, LIMK1, PBK, CDC25C, MAD2L1, HMGA2, GRIN3A, and IGF1 had widespread deletion frequencies (Fig. 1D). Moreover, the CNV alterations of these prognostic MCRGs were located on chromosomes (Fig. 1E). The landscape showed that 114 patients (25.3%) displayed genetic mutations, and the most common variant was missense mutation, mainly C > T mutation (Fig. 1F).

### Development of an MC-related prognostic gene model in the TCGA cohort

**Figure 2:**
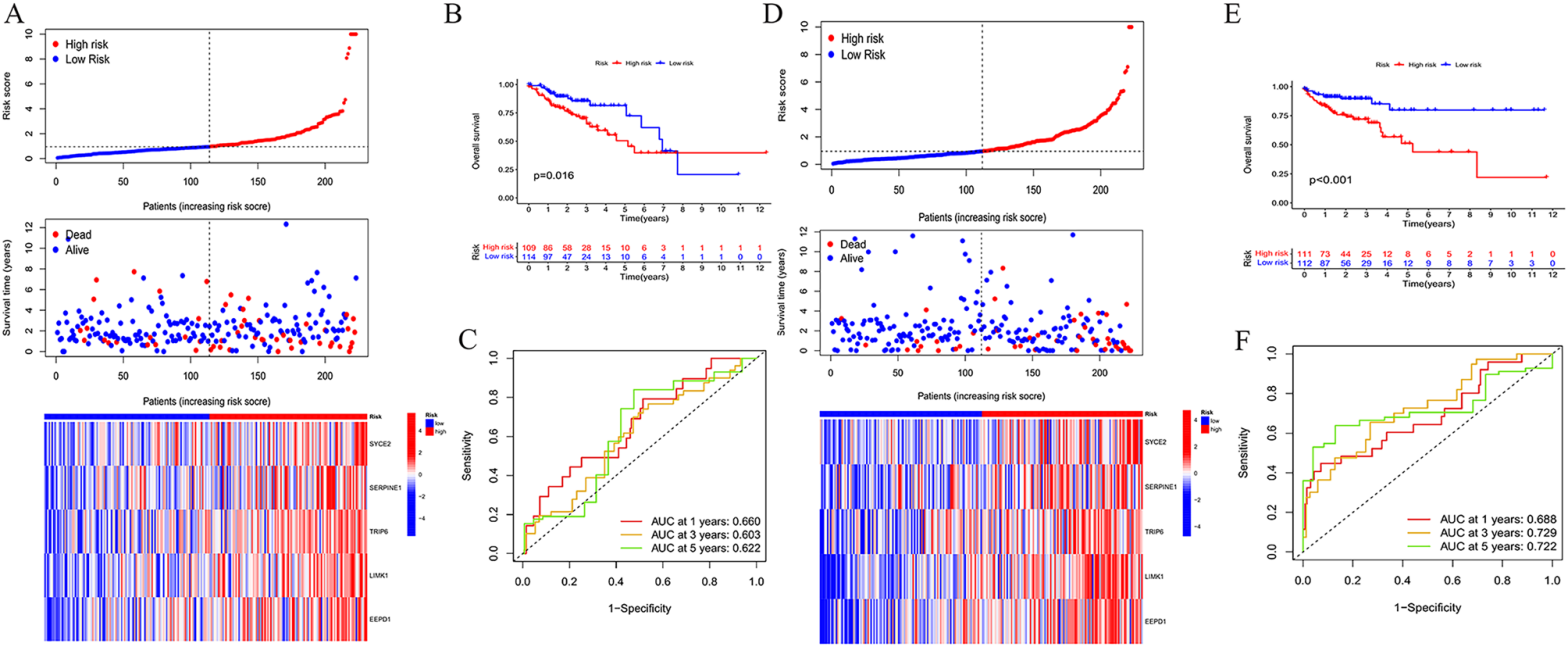
Construction and internal validation of a prognostic model for MCRGs. (**A**) Heat map of risk score distribution, survival status and MCRG expression for high-risk and low-risk patients in the training set. (**B**) Survival curves of high- and low-risk patients in the training set. (**C**) ROC curves for the prognostic model of MCRGs in the training set predicting overall survival at 1, 3 and 5 years. (**D**) Heat map of risk score distribution, survival status and MCRG expression for high-risk and low-risk patients in the test set. (**E**) Survival curves of high- and low-risk patients in the test set. (**F**) ROC curves for the prognostic model of MCRGs in the test set predicting overall survival at 1, 3 and 5 years.

In the TCGA cohort, we developed a prognostic gene model related to MC by identifying five hub genes (SYCE2, SERPINE1, TRIP6, LIMK1, and EEPD1) with prognostic value through LASSO analysis (Fig. S1). We used the formula Risk score = 0.972240964SYCE2 + 0.208393835SERPINE1 + 0.244958765TRIP6 + 0.583762591LIMK1 + 0.511535266 * EEPD1 to divide the 446 COAD samples into high-risk and low-risk populations. The training and testing sets confirmed that the survival time was decreased as the risk scores of these five genes increased (Fig. 2A,D). Additionally, the Kaplan–Meier curve showed that high-risk scores had worse overall survival probability than those with low-risk scores (Fig. 2B,E), and ROC curves predicted the sensitivity and specificity of the MCRGs signature, which reached 0.660, 0.603, 0.622 (in the training set), and 0.688, 0.729, 0.722 (in the test set) in 1-, 3-, and 5-year, respectively (Fig. 2C,F). In the entire set, fewer deaths were observed in the low-risk group, and the prognosis of the low-risk group was better than that of the high-risk group (Fig. S2A,B). ROC curves reached 0.674, 0.662, and 0.692 for 1-, 3-, and 5-year survival, respectively, demonstrating the predictive performance of the risk signature in the entire set (Fig. S2C).

### Nomogram construction and validation

**Figure 3:**
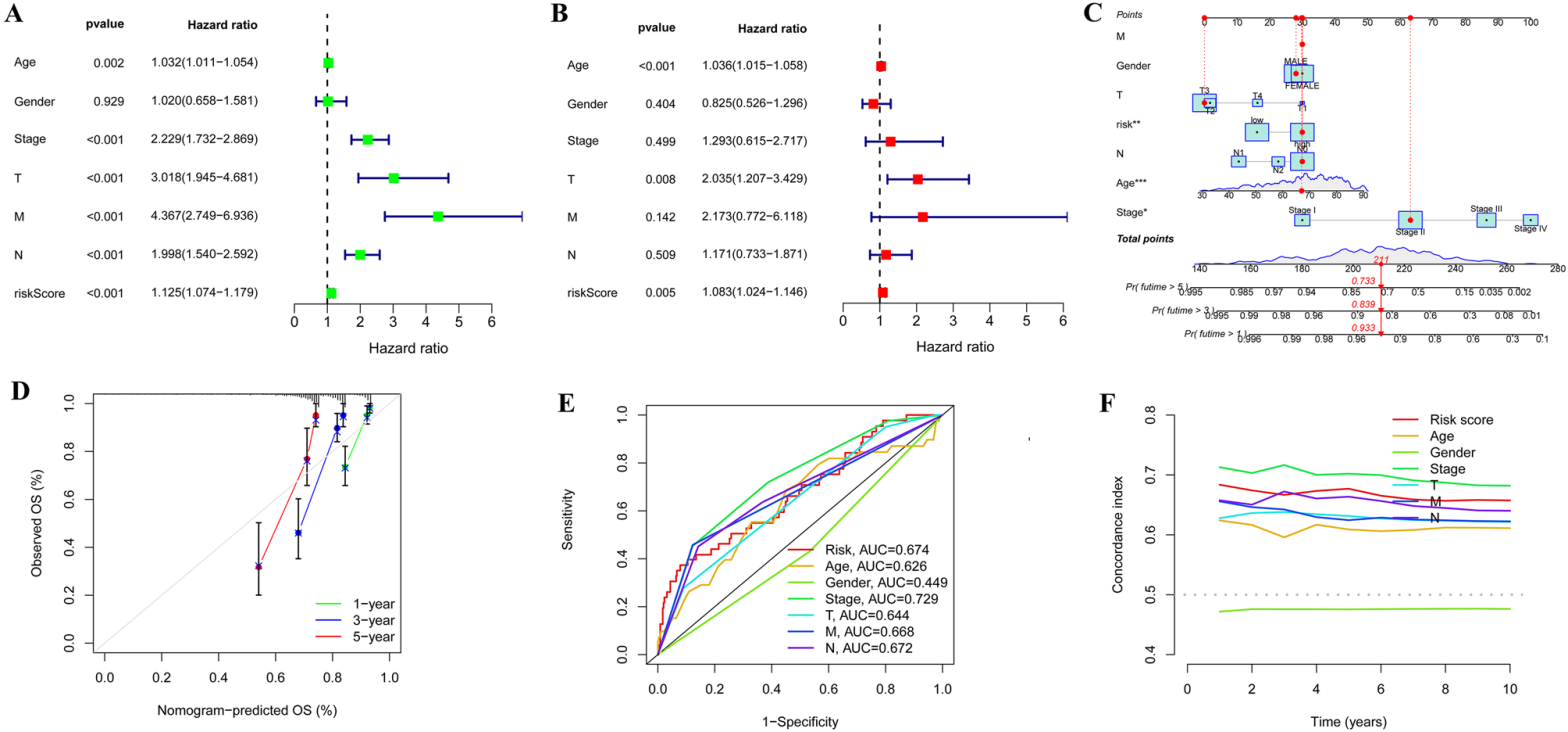
Construction of predictive nomogram. (**A**–**B**) Forest plot of univariate and multivariate Cox regression analysis of patient prognosis. (**C**–**D**) A designed nomogram predicted overall patient survival at 1, 3, and 5 years and calibration curves. (**E**) The ROC curves show the prediction efficiency of the nomogram in the TCGA cohort. (**F**) C-index for age, sex, stage, T-stage, M-stage, N-stage, and risk score.

**Figure 4:**
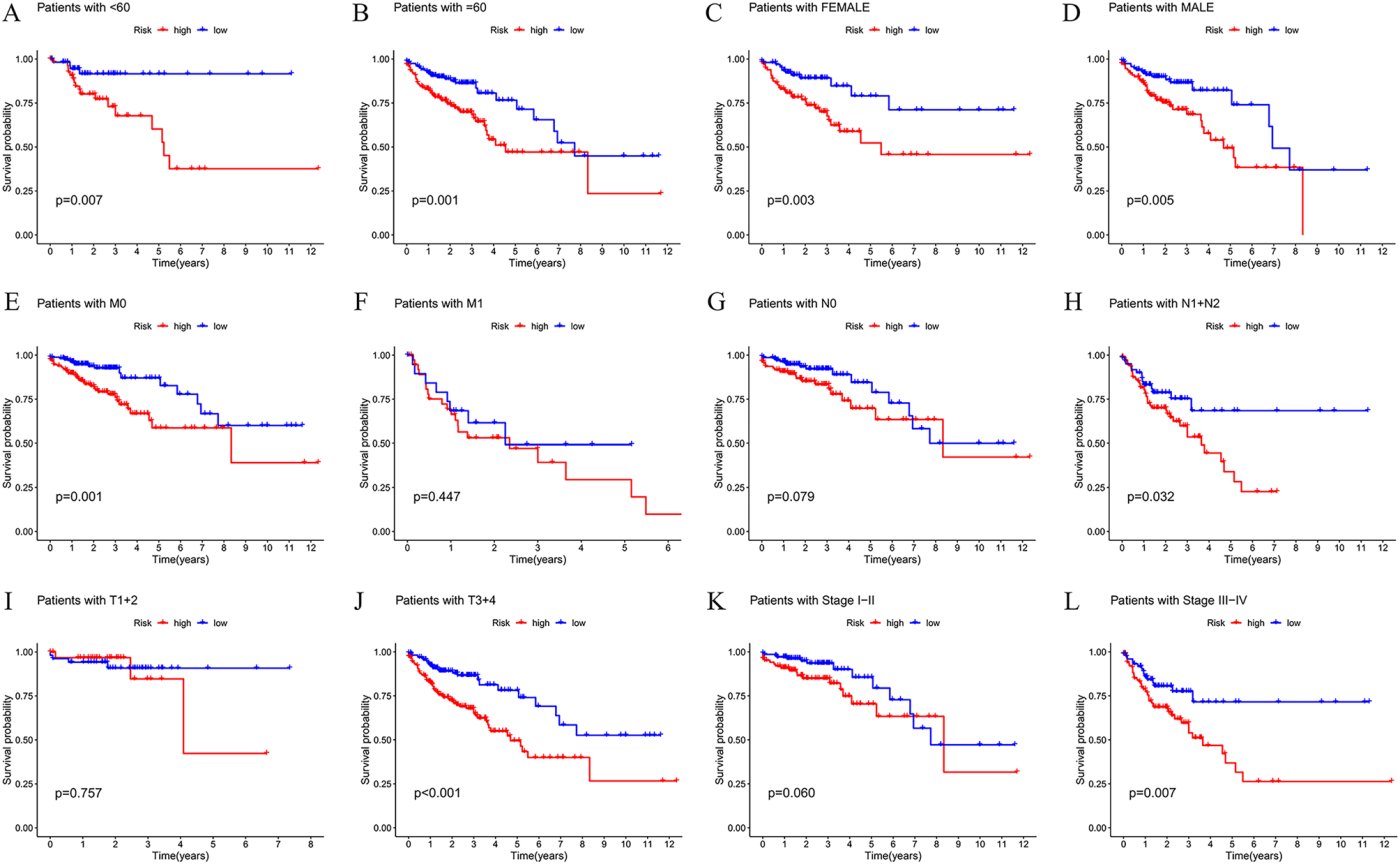
Analysis of survival differences between high- and low-risk population subgroups in the TCGA cohort. OS analysis of risk scores in high-risk and low-risk patients with patient age > 60 years (**A**), <  = 60 years (**B**), female (**C**), male (**D**), M0 stage (**E**), M1 stage (**F**), N0 stage (**G**), N1 + N2 stage (**H**), T1 + 2 stage (**I**), T3 + 4 stage (**J**), I–II stage (**K**), and III–IV stage (**L**).

We conducted univariate and multivariate Cox regression analyses on the TCGA dataset, which revealed that our risk signature and clinical variables such as age, stage, T stage, M stage, and N stage were independent prognostic factors that affected COAD patients (Fig. 3A–B). To validate our findings, we designed a nomogram that displayed relatively good predictions for the 3-year and 5-year overall survival rates in the entire cohort (Fig. 3C–D). We also evaluated the performance of our signature by calculating the AUC values of various clinical factors, including age, stage, T stage, M stage, and N stage, and found that our signature had a higher C-index advantage (Fig. 3E–F). These results indicated that our MCRGs signature was a superior clinical target for predicting the OS of COAD patients. Furthermore, our survival analysis of clinical characteristics based on the MCRGs showed that our signature could significantly distinguish patients with different age groups, genders, M and N stages, T stages, and stages III + IV (Fig. 4, *P* < 0.05), suggesting that our model has the potential to predict different clinical signs.

### Characteristic genotype analysis

**Figure 5:**
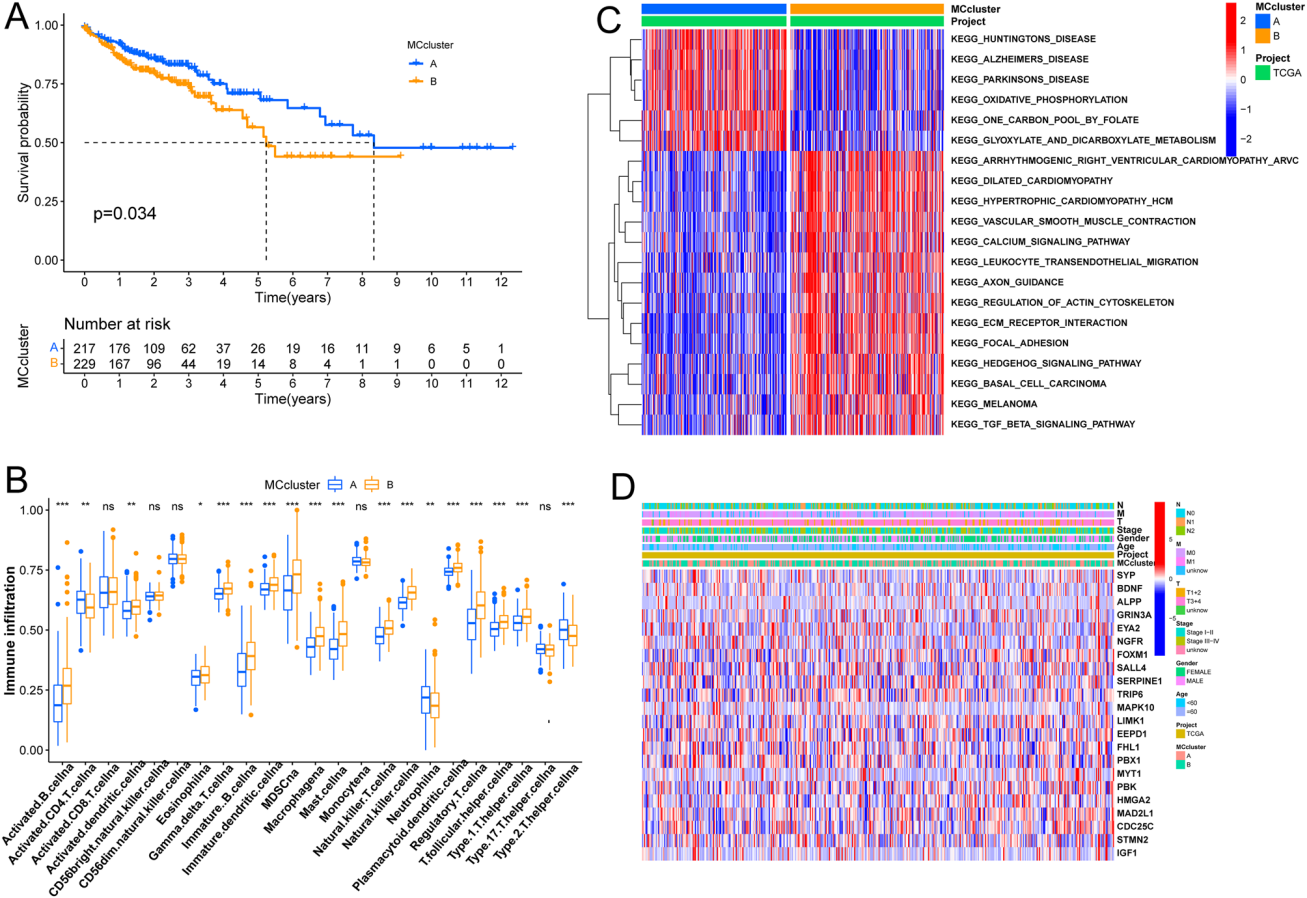
Defining molecular subtypes of COAD patients and their characteristics based on 5 MCRGs (**A**) OS survival curves were analyzed in 2 different subtypes of patients. (**B**) Differences in infiltration scores of 23 immune markers in high and low risk groups based on ssGSEA algorithm. (**C**) GSVA enrichment analysis of 2 COAD subtypes. (**D**) Clinicopathological characteristics of 2 different subtypes and heat map of DEPRGs expression. *P < 0.05, **P < 0.01, ***P < 0.001, ns, no significance.

Based on the selected k value and the cophenetic correlation coefficient, we classified all patients with COAD into two subtypes (clusters A and B) (Fig. S3A–C), which were then further analyzed using Principal component analysis (PCA) to reveal the subtypes based on MCRGs (Fig. S3D). Results showed that patients in cluster B had worse overall survival in COAD than those in cluster A (Fig. 5A), and the differences in immune cell fraction between the two clusters were further highlighted by the boxplot (Fig. 5B). GSVA analysis revealed that cluster A was enriched in huntingtons disease, alzheimers disease, and oxidative phosphorylation, while cluster B was enriched in arrhythmogenic right ventricular cardiomyopathy (ARVC), dilated cardimyopathy, hypertrophic cardiomyopathy (HCM), and calcium signaling pathway (Fig. 5C). In addition, based on clinical features (N stage, M stage, T stage, stage, gender, age and MC cluster), we constructed a heatmap of the MCRGs that took into account the complex cluster and risk score (Fig. 5D).

### Immune function, enrichment analysis, and risk signature variability

**Figure 6:**
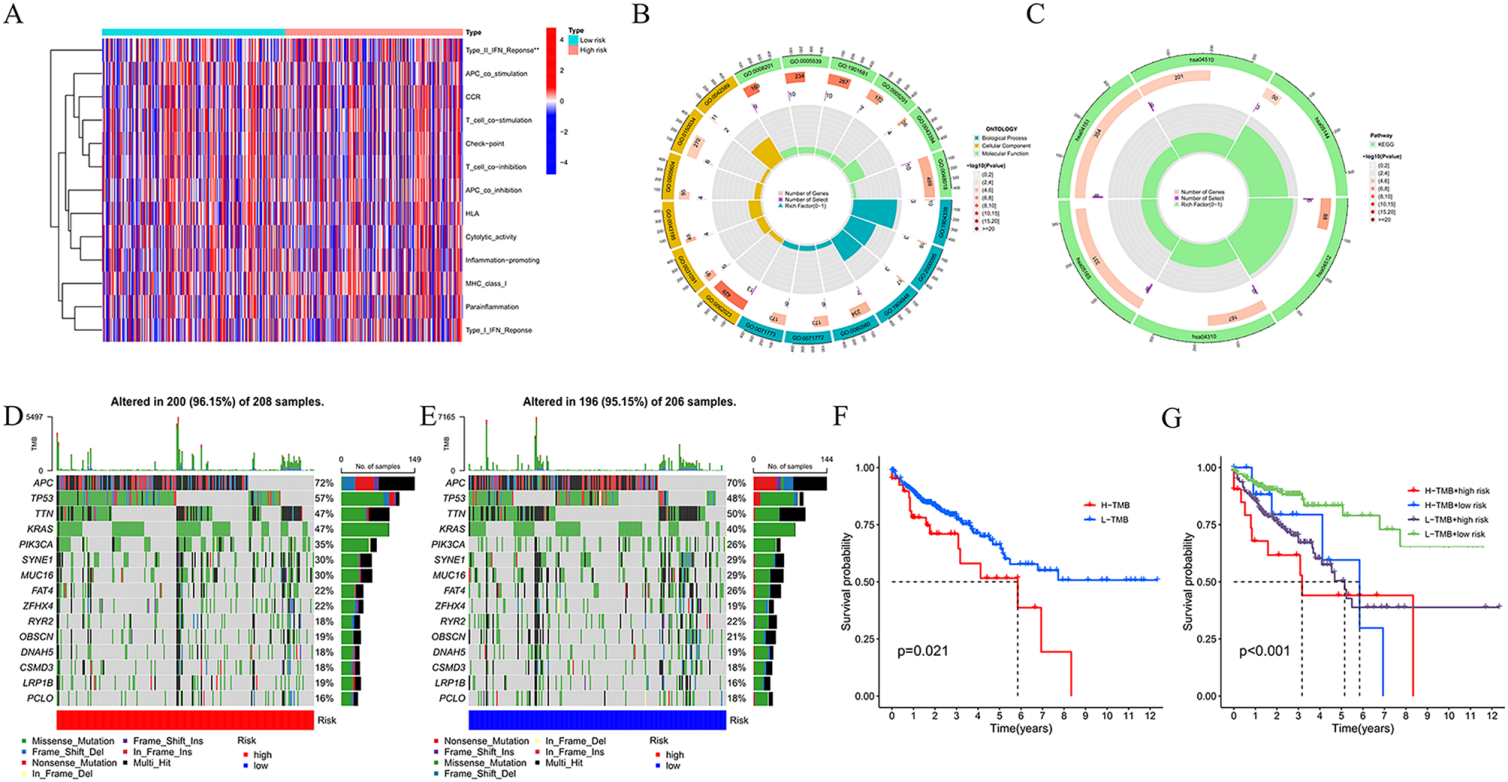
Mechanisms related to MCRG risk signature in COAD. (**A**) Heat map of immune cell inflammation in high- and low-risk populations. (**B**) Gene Ontology (GO). (**C**) KEGG analysis. (**D**) Frequency and type of gene mutations in high-risk group. € Frequency and type of gene mutations in the low-risk group. (**F**) OS survival curve analysis in low TMB group and high TMB group. (**G**) OS survival curve analysis between different TMB subgroups in high and low risk groups.

To comprehensively explore the immune function between different risk groups and immune cell infiltration, we generated a heatmap (Fig. 6A). Additionally, we performed Gene Ontology (GO) enrichment analysis to explore the underlying molecular mechanisms of the MCRGs-based model, which mainly involved cell differentiation and cell polarity (Fig. 6B). Through KEGG analysis, we found that the model was mainly involved in ECM-receptor interaction, Wnt signaling pathway, Human papillomavirus infection, PI3K-Akt signaling pathway, and Focal adhesion (Fig. 6C).

Through the waterfall plots, we revealed that the mutation profiles of high-risk patients were higher than those of the low-risk group, except for TTN (Fig. 6D–E).

Based on the TMB scores, we detected the prognosis and found that high TMB scores were associated with worse survival (Fig. 6F). Furthermore, we validated that the MC risk signature was better at predicting OS outcomes than TMB scores. Patients with high and low TMB scores in the high-risk groups (H-TMB of high risk and L-TMB of high risk) displayed poorer OS than patients with high- and low-TMB scores in the low-risk groups (Fig. 6G).

### The protein expression of 5 hub MCRGs and prognostic evaluation

**Figure 7:**
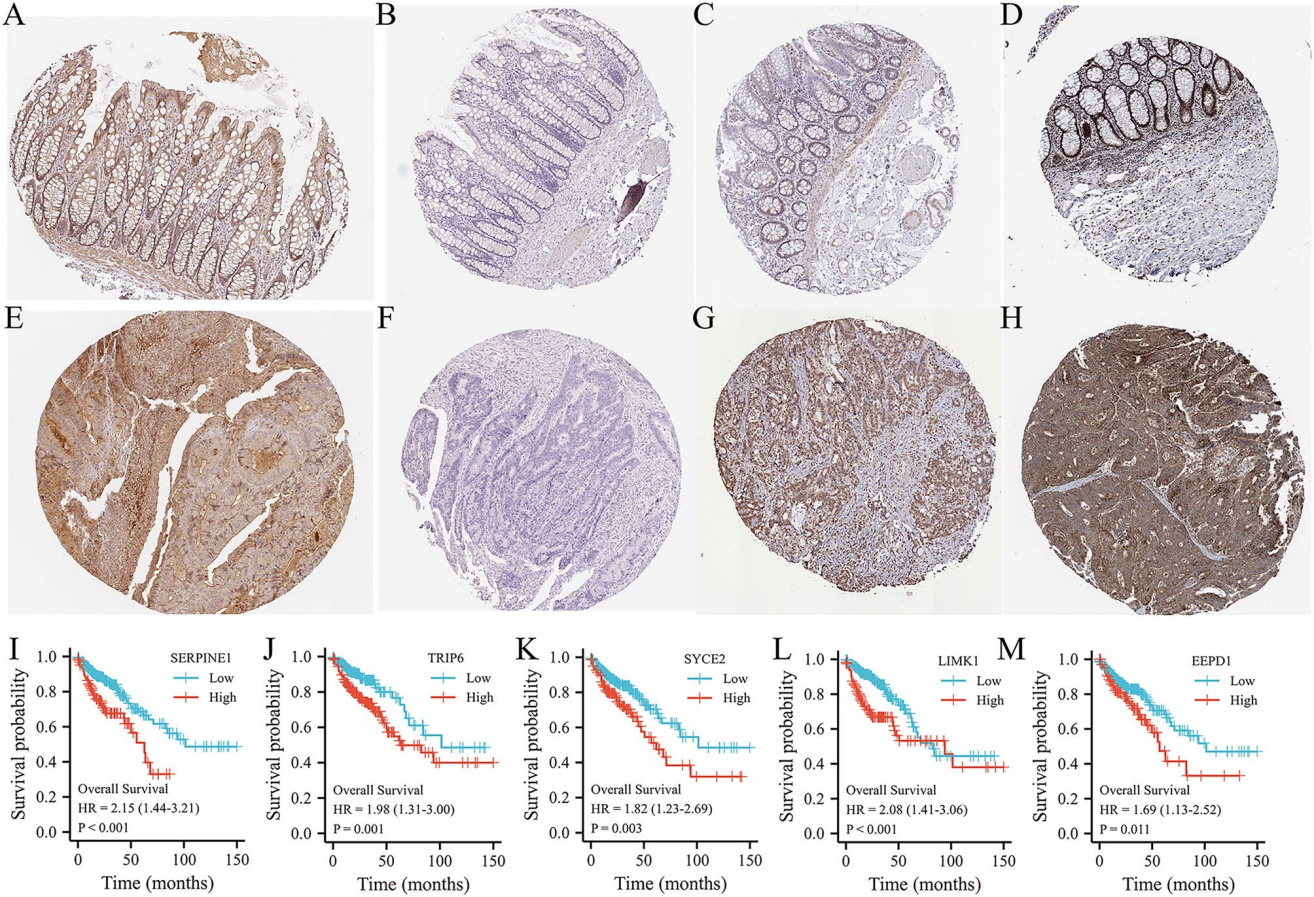
The expression of 4 MCRGs from risk signatures in HPA databse. (**A**–**D**) Immunohistochemistry of 4 MCRGs (SYCE2, SERPINE1, TRIP6 and LIMK1) in COAD tissues^43^. Immunohistochemistry of 4 MCRGs (SYCE2, SERPINE1, TRIP6 and LIMK1) in normal colon tissues. (**I**–**M**) Survival curve analysis of 5 MCRGs in Kaplan–Meier Plotter.

Through HPA database for immunohistochemical data of SYCE2, SERPINE1, TRIP6, and LIMK1, and EEPD1. We found that these genes were all highly expressed in COAD tissues (Fig. 7A–D) in comparing with normal tissues (Fig. 7E–H). Then, we used the Kaplan–Meier Plotter website to verify the prognostic value of the 5 hub genes. The expression of these 5 genes were related to inferior OS (Fig. 7I–M).

### Relationships between MCRGs and chemotherapy drug sensitivity

**Figure 8:**
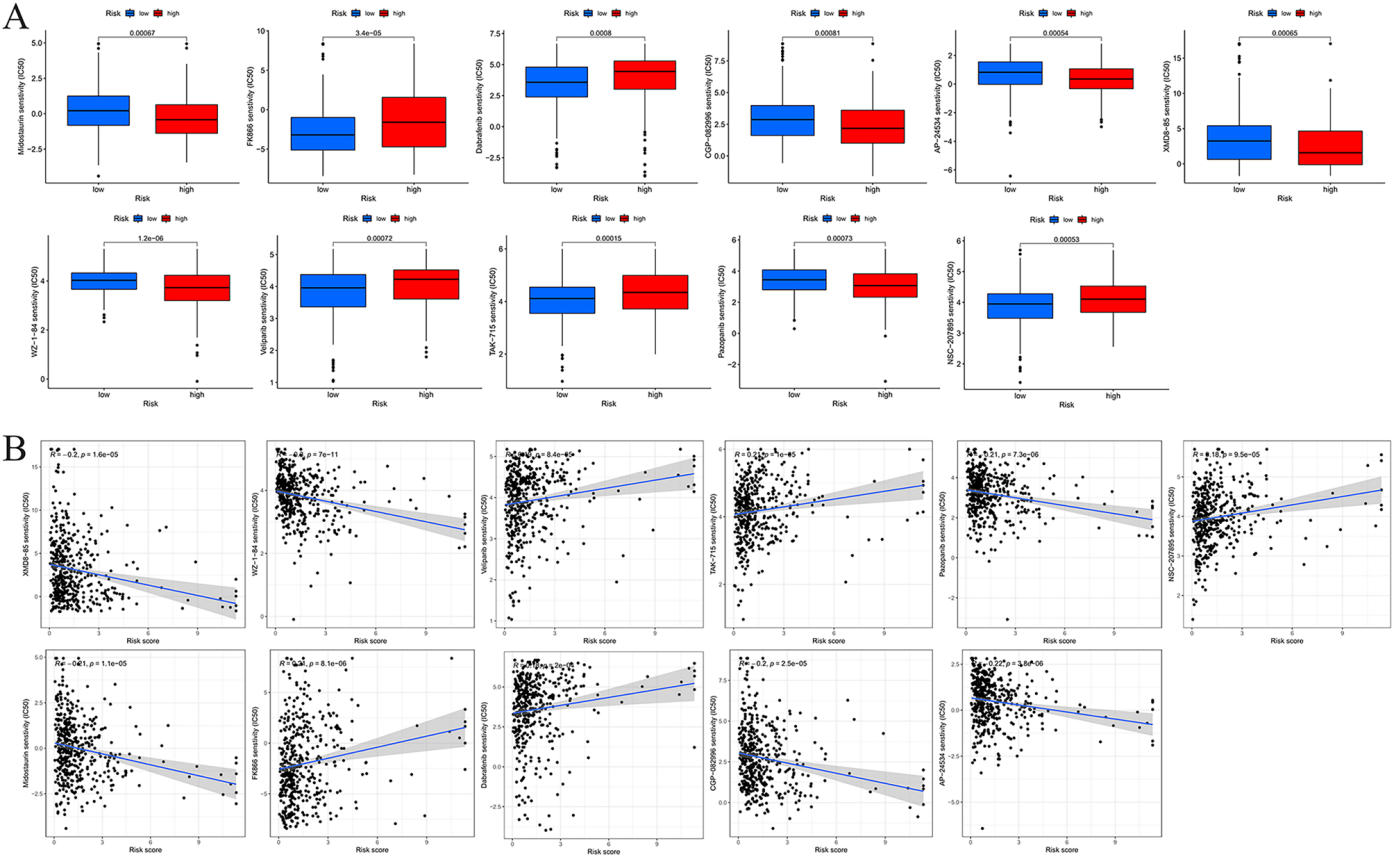
Drug sensitivity analysis. (**A**) Differences in IC50 of AP-24534, CGP-082996, Dabrafenib, FK866, Midostaurin, NSC-207895, Pazopanib, TAK-715, Veliparib, WZ-1-84 and XMD8-85 in high and low risk groups. (**B**) Correlation analysis of MCRG risk score and drug IC50.

Figure 8A illustrated the results of the drug sensitivity test. Potential chemotherapy drugs were identified by measuring the IC50 between the low-risk and high-risk groups. COAD patients in the high-risk subtype were evidently sensitive to chemotherapy drugs such as AP-24534, CGP-082996, Dabrafenib, FK866, Midostaurin, NSC-207895, Pazopanib, TAK-715, Veliparib, WZ-1–84, and XMD8-85 (all *P* < 0.05). While low-risk groups were sensitive to FK866, Veliparib, Dabrafenib, TAK-75 and NSC-207895. Moreover, we confirmed the association between the risk signature and chemotherapy drugs (Fig. 8B).

### MCRGs expression was high in CRC tissues

**Figure 9:**
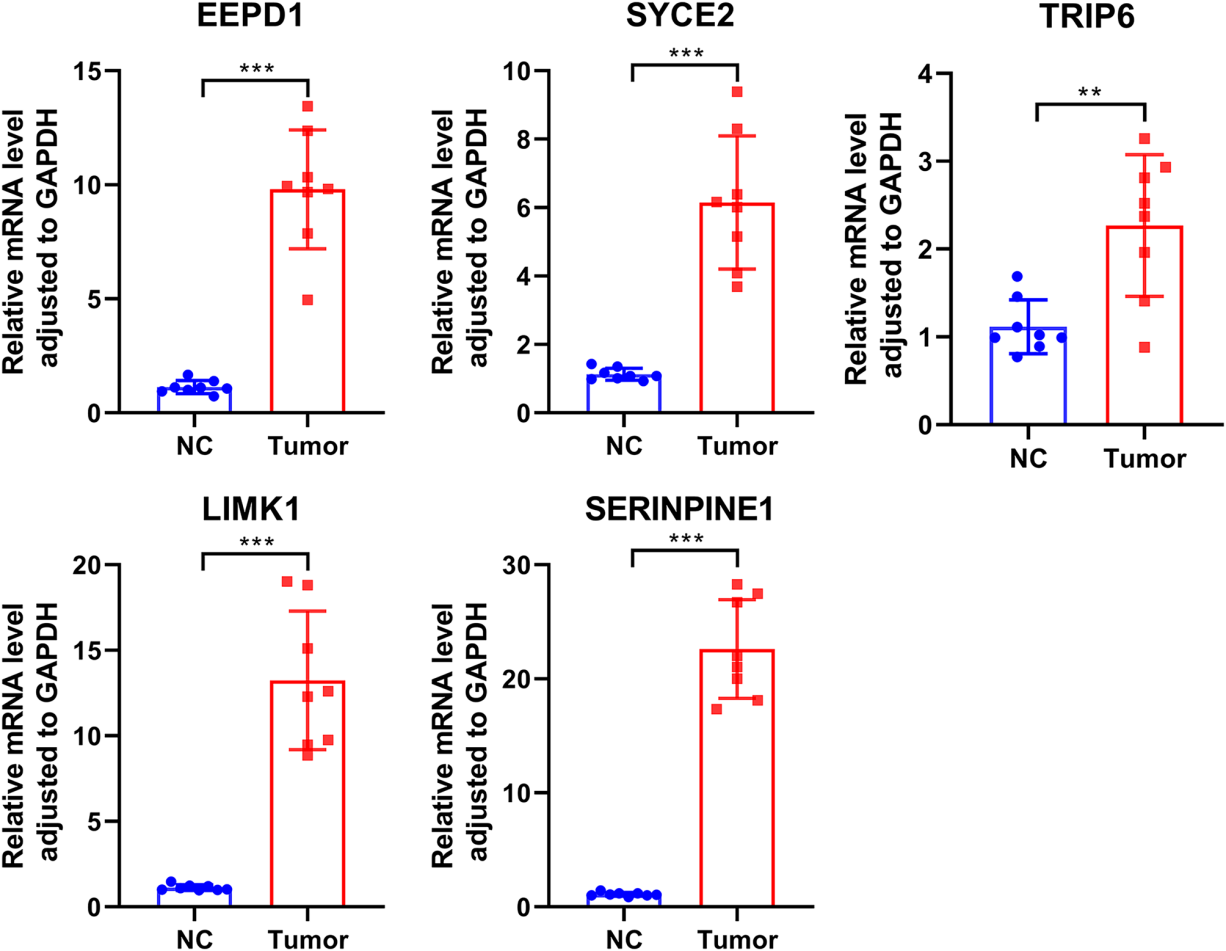
The expression levels of the oncogenes (SYCE2, SERPINE1, TRIP6, LIMK1 and EEPD1) were up-regulated in CRC tissues as shown by qRT-PCR results (n = 8). ** P ≤ 0.01, *** P ≤ 0.001. The results were presented as mean ± SEM.

To validate the expression profile of MCRGs in clinical patients, we examined the expression of oncogenes (SYCE2, SERPINE1, TRIP6, LIMK1, and EEPD1) by RT-qPCR in 8 pairs of clinical samples from CRC patients. According to the qPCR results, the oncogenes were expressed at high levels in CRC tissues (Fig. 9).

## Discussion

As a malignancy with high mortality rate and unfavorable prognosis, the development of colon adenocarcinoma (COAD) can be influenced by various factors^3^. Among them, (MCRGs have been shown to be essential in the prevention, treatment, and drug resistance of many cancers, including COAD^11,12,27,28^. However, the study of MCRGs in cancer development has been limited to individual MCRGs, and there is a lack of systematic and comprehensive exploration of the combined effects of a large number of MCRGs on cancer^14,29,30^. In this study, we screened 23 prognosis-associated MCRGs in the TCGA-COAD cohort and revealed variations of MCRGs in COAD at the transcriptional and genetic levels. Then, we developed a risk prognostic model consisting of five MCRGs in combination with the survival status of COAD patients. This model could independently predict the survival status of patients with different clinicopathological features and revealed the underlying mechanisms and associated factors, such as the immune microenvironment and mutational status. The drug sensitivity analysis confirmed the association between risk characteristics and chemotherapeutic agents, potentially providing a basis for more precise application of relevant drugs.

Mitotic catastrophe is a regulated anti-proliferative process that occurs during defective or failed mitosis, and is an oncosuppressive mechanism that maintains genomic stability^10^. It remains unknown how MCRGs are expressed in COAD and whether they are associated with survival time in patients. Based on the expression of MCRGs, screening for biomarkers with prognostic value is expected, which could provide a reliable assessment tool for COAD patients with poor prognosis. Using LASSO Cox regression analysis, an independent prognostic gene model based on five MCRGs (SYCE2, SERPINE1, TRIP6, LIMK1, and EEPD1) was constructed to predict the overall survival^25^ of COAD patients with different clinicopathological characteristics. Surprisingly, many MCRG-based prognostic models have not been developed so far. The 5 prognostic MCRGs identified in our study have been mentioned in relevant prognostic studies. For example, Ye et al.^31^ reported a 9-gene prognostic model including SYCE2 in gastric cancer, which was able to accurately predict the overall survival of gastric cancer patients. High expression of SERPINE1 is significantly associated with poor prognosis in various cancers, including COAD^32^, gastric cancer^33,34^, ovarian cancer^25^, and breast cancer^35^. Similarly, Wei et al.^36^ developed a 6-gene prediction model including SERPINE1 to predict the survival outcome of breast cancer patients at 1, 3, and 5 years, and its accuracy and reliability were validated. During tumorigenesis and progression, studies have confirmed that LIMK1 is upregulated in various tumors, is associated with patient prognosis, and is closely associated with changes in the biological behavior of several human tumors^37–39^. Recent findings also suggest that LIMK1 may be a valuable and promising biomarker for the diagnosis of CRC^39^. EEPD1 is also overexpressed in various solid tumors, including colon cancer, but related studies have focused more on its role in DNA damage^40^. It was found that DNA damage may lead to mitotic catastrophe and that tumor cells are more susceptible to mitotic abnormalities than normal cells^41^. These examples evince that our findings are consistent with prior studies. Interestingly, we also divulge novel discoveries that lay the groundwork for future MCRG studies in COAD.

The signature prognostic model stemming from our study significantly correlated with clinical traits, immune cells, TMB, cancer-related pathways, and drug sensitivity. To our knowledge, this is the first comprehensive analysis of the connection between MC, gene mutations, and the immune microenvironment in COAD. The growth of cancer is influenced by multiple factors and various genes. A composite multigene prognostic model will furnish an optimized risk score using regression methods. The ROC method demonstrated that risk scores have high sensitivity and specificity as prognostic factors. The nomogram also signifies that it is a critical independent prognostic risk factor among clinical variables. Our results showed that samples were adequately distributed into two independent subtypes and significantly linked with survival, with notable variances in the immune microenvironment regarding the proportion of some immune cells between the two subtypes, such as activated B cell, CD4+ T cell, MDSC, among others. Few studies have delved into the correlation between MCRG and immunotherapy. Nevertheless, several prognostic models have been scrutinized concerning the immune microenvironment. For instance, SERPINE1 is linked with the reconstruction of the tumor microenvironment and infiltration of immune cells in the progression of colon cancer^42^. Moreover, LIMK1 is also related to multiple tumor-infiltrating immune cells in CRC, notably CD4+ T cells and macrophage^39^.

Additionally, the cancer-associated genomes between high- and low-risk scoring groups are profoundly involved in cellular processes, furnishing insights into MCRG-linked mechanistic alterations in COAD. The GO- and KEGG-enriched 5-gene marker pathway was substantially associated with the development and progression of colon cancer, proposing that this marker could be utilized as a prognostic marker for clinical diagnosis. Our study showed high mutations in the high-risk group and poor prognosis in patients with high tumor mutation burden, especially in patients with high risk scores, suggesting crosstalk between mutations and MCRG in COAD. Mitotic catastrophes are entangled in the antitumor effects of various chemotherapeutic agents, including microtubule modulators, CHK1 inhibitors, PARPs inhibitors, WEE1 inhibitors, PLKs inhibitors, among others. We confirmed the correlation between CMCRG risk profile and chemotherapeutic agents. Mitotic catastrophe is a popular target for the development of novel anticancer drugs, particularly considering its avoidance for tumor resistance. On the one hand, tumor cells typically have an abnormal genome number, making them particularly susceptible to mitotic catastrophe-inducing drugs. On the other hand, some currently available chemotherapy regimens that induce apoptosis have been discovered to be effective in triggering mitotic catastrophe at lower doses.

## Conclusion

In conclusion, our study revealed a 5-gene signature associated with the prognosis of colon cancer patients that can serve as a potentially reliable prognostic biomarker and therapeutic target for COAD patients with different clinicopathological features, and potentially provide a basis for more precise application of relevant drugs.

### Supplementary Information

Supplementary Legends.

Supplementary Figure S1.

Supplementary Figure S2.

Supplementary Figure S3.

Supplementary Table S1.

Supplementary Table S2.

Supplementary Table S3.

## Data Availability

Publicly available datasets were analyzed in this study. These data can be found here: all relevant raw data used in the study can be accessed from TCGA (https://portal.gdc.cancer.gov/repository) and GENCARDS (https://www.genecards.org/).
