# Author Correction: Developing a prognosis and chemotherapy evaluating model for colon adenocarcinoma based on mitotic catastrophe-related genes

**DOI:** 10.1038/s41598-024-54131-8

**Published:** 2024-02-27

**Authors:** Yinglei Liu, Yamin Zhao, Siming Zhang, Shen Rong, Songnian He, Liqi Hua, Xingdan Wang, Hongjian Chen

**Affiliations:** 1 Nantong Tumor Hospital and Affiliated Tumor Hospital of Nantong University, Nantong, China; 2 Affiliated Hospital 2 of Nantong University, Nantong First People’s Hospital, Nantong, China; 3 The Second People’s Hospital of Nantong, Nantong, China

Correction to: *Scientific Reports* 10.1038/s41598-024-51918-7, published online 18 January 2024

In the original version of this Article, the Affiliations were not listed in the correct order. The correct affiliations are listed below:

Affiliation 1:

Nantong Tumor Hospital and Affiliated Tumor Hospital of Nantong University, Nantong, China.

Affiliation 2:

Affiliated Hospital 2 of Nantong University, Nantong First People’s Hospital, Nantong, China.

Affiliation 3:

The Second People’s Hospital of Nantong, Nantong, China.

The original Article has been corrected.

