# Supplementary material for: Developing a prognosis and chemotherapy evaluating model for colon adenocarcinoma based on mitotic catastrophe-related genes
Source: Sci Rep. 2024 Jan 18;14:1655. doi: 10.1038/s41598-024-51918-7 (PMC10796338; doi:10.1038/s41598-024-51918-7)
Supplement: Supplementary file 1 — Supplementary Legends. [file 41598_2024_51918_MOESM1_ESM.docx]

Supplementary Tab. S1 The obtained MCRGs from GENCARDS database.

Supplementary Tab. S2 Expression levels of MCRGs in TCGA cohort for differential analysis.

Supplementary Tab. S3 Expression levels of prognostic-related MCRGs in TCGA cohort for univariate Cox regression analysis.

Supplementary Fig. S1Least absolute shrinkage and selection operator (LASSO) regression analysis based on 5 prognostic MCRGs in the TCGA cohort.

(A) LASSO regression of the OS-related MCRGs. (B) Error rate plot for cross-validation in LASSO regression.

Supplementary Fig. S2 Prognostic model characteristics of the entire set of MCRGs

(A) Heat map of risk score distribution, survival status and MCRG expression for high-risk and low-risk patients in the entire set. (B) Survival curves of high- and low-risk patients in the entire set. (C) ROC curves for the prognostic model of MCRGs predicting overall survival at 1, 3 and 5 years.

Supplementary Fig. S3 Consensus clustering analyses for COAD subtypes.

(A-B) Cumulative Distribution Function (CDF) Curves and Delta Area Plot for k=2-9 in the Consistent Clustering Model. (C) Consistency clustering matrix heat map (k=2). (D) PCA analysis revealed differences in transcriptome data distribution between the 2 COAD isoforms.
