# Supplementary figures and images for: Developing a prognosis and chemotherapy evaluating model for colon adenocarcinoma based on mitotic catastrophe-related genes
Source: Sci Rep. 2024 Jan 18;14:1655. doi: 10.1038/s41598-024-51918-7 (PMC10796338; doi:10.1038/s41598-024-51918-7)

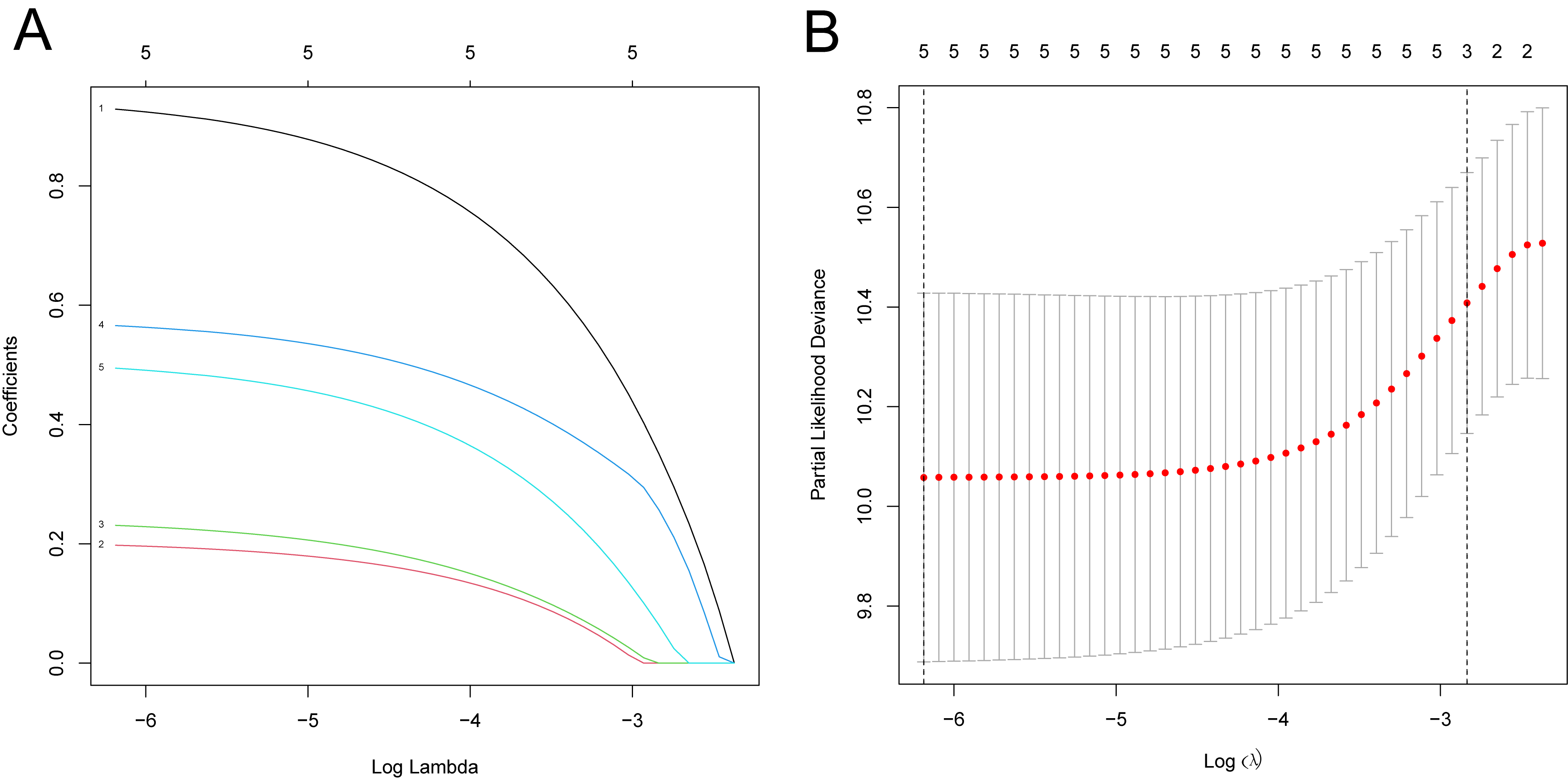

Supplement: Supplementary file 2 — Supplementary Figure S1. [file 41598_2024_51918_MOESM2_ESM.tif]

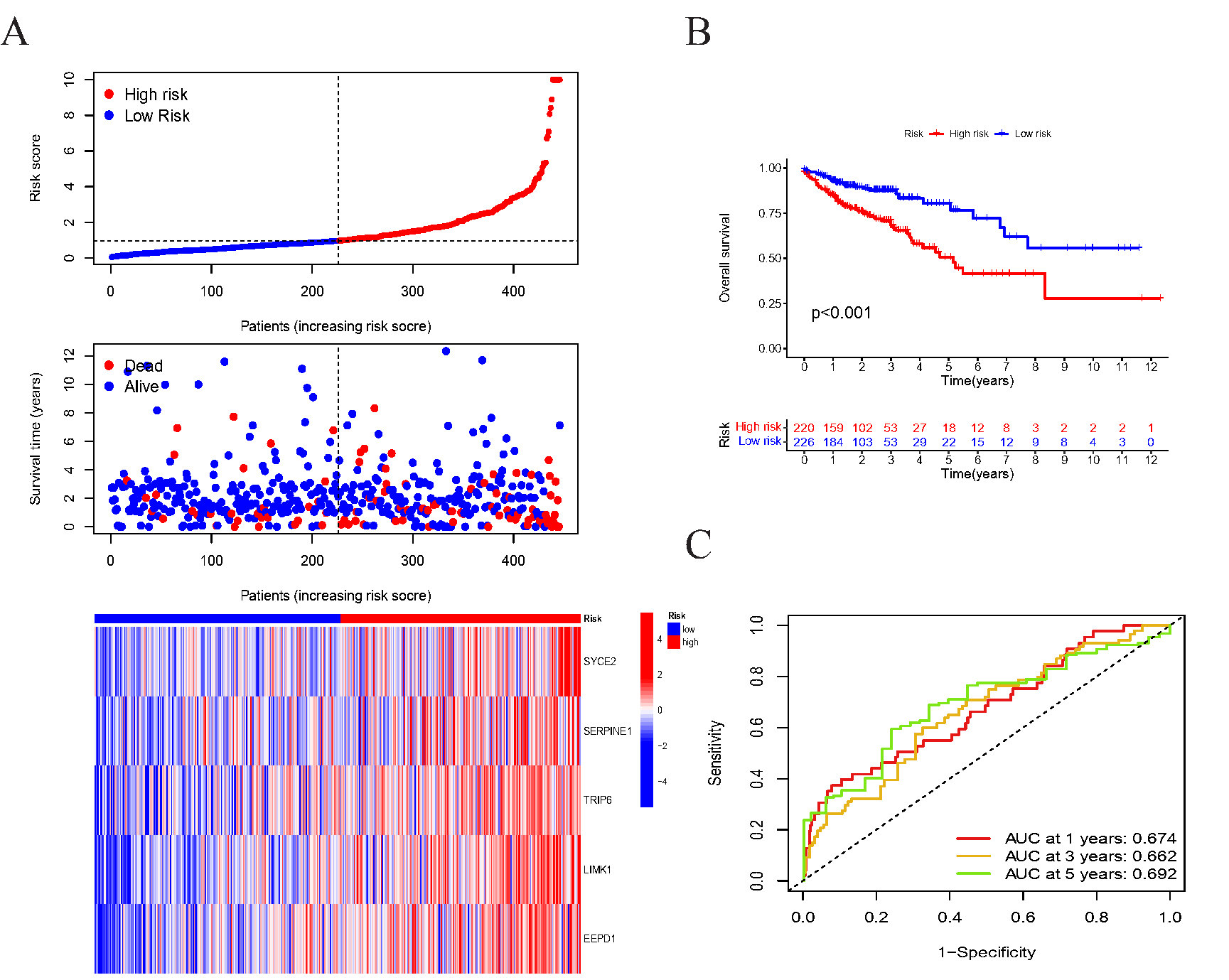

Supplement: Supplementary file 3 — Supplementary Figure S2. [file 41598_2024_51918_MOESM3_ESM.jpg]

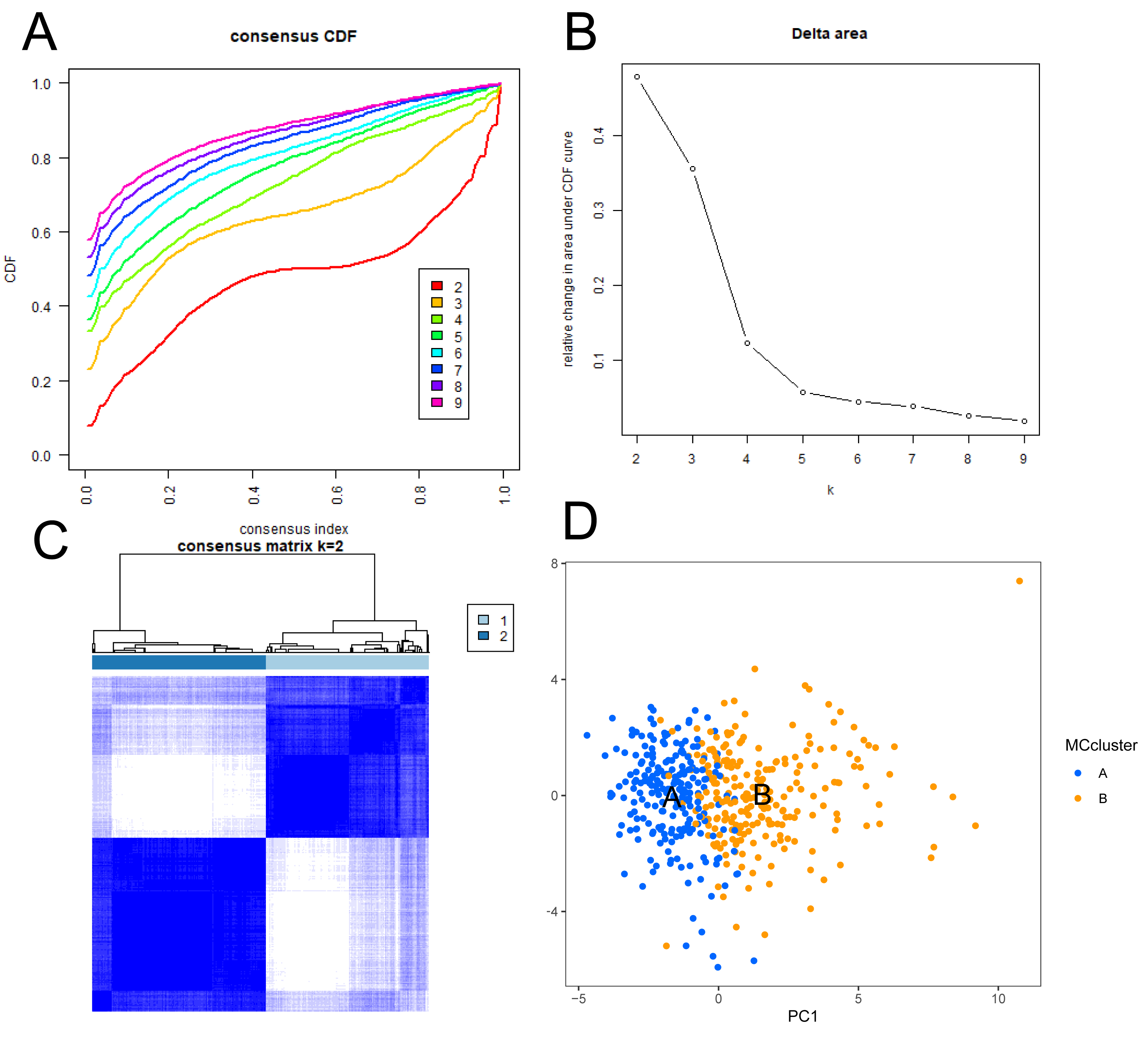

Supplement: Supplementary file 4 — Supplementary Figure S3. [file 41598_2024_51918_MOESM4_ESM.tif]
